# Is Radon Emission in Caves Causing Deletions in Satellite DNA Sequences of Cave-Dwelling Crickets?

**DOI:** 10.1371/journal.pone.0122456

**Published:** 2015-03-30

**Authors:** Giuliana Allegrucci, Valerio Sbordoni, Donatella Cesaroni

**Affiliations:** Department of Biology, University of Rome Tor Vergata, Rome, Italy; University of Cincinnati, UNITED STATES

## Abstract

The most stable isotope of radon, ^222^Rn, represents the major source of natural radioactivity in confined environments such as mines, caves and houses. In this study, we explored the possible radon-related effects on the genome of *Dolichopoda* cave crickets (Orthoptera, Rhaphidophoridae) sampled in caves with different concentrations of radon. We analyzed specimens from ten populations belonging to two genetically closely related species, *D*. *geniculata* and *D*. *laetitiae*, and explored the possible association between the radioactivity dose and the level of genetic polymorphism in a specific family of satellite DNA (*pDo500* satDNA). Radon concentration in the analyzed caves ranged from 221 to 26000 Bq/m^3^. Specimens coming from caves with the highest radon concentration showed also the highest variability estimates in both species, and the increased sequence heterogeneity at *pDo500* satDNA level can be explained as an effect of the mutation pressure induced by radon in cave. We discovered a specific category of nuclear DNA, the highly repetitive satellite DNA, where the effects of the exposure at high levels of radon-related ionizing radiation are detectable, suggesting that the satDNA sequences might be a valuable tool to disclose harmful effects also in other organisms exposed to high levels of radon concentration.

## Introduction

Radon is a radioactive gas occurring naturally. It is part of the normal radioactive chain of uranium and represents the decay product of radium. It is a rare gas and usually migrates freely through faults and fragmented soils and may accumulate in caves and / or water. The most stable isotope of radon, ^222^Rn, has a half-life of about 4 days and due to this characteristic, its concentration decreases with increasing distance from the production area. Ground water has generally higher concentrations of ^222^Rn than surface water because the radon is continuously produced by the radium present in the rocks. ^222^Rn can be significantly high in hot sulfur spring waters [1]. Due to these characteristics, ^222^Rn represents the major source of natural radioactivity in confined environments such as mines, caves and houses. Typical domestic exposures are about 100 Becquerel per cubic meter (Bq/m^3^) indoors and 10–20 Bq/m^3^ outdoors [2]. Concentration limits of radon for domestic areas are variable and depend on the organization; the European Union established two threshold values, one for the old houses (400 Bq/m^3^) and one for the new ones (200 Bq/m^3^), while the US-EPA (2007) put the limit at concentration of 74 Bq/m^3^. Studies have demonstrated a significant and dose-related excess of lung cancer in radon-exposed miners (National Research Council 1988) and several ecologic studies have found increased rates of leukaemia in regions with elevated levels of radon in homes [3,4, 5, 6,7].

In caves, radon concentration is known to vary within an extremely wide range [8, 9]. Natural caves of volcanic origin can be characterized by exceedingly high levels of radon because of the presence of uranium and therefore of the decay chain products of uranium series [10, 11]. Artificial caves as cellars, Etruscan graves, and Roman cisterns are often built with tuff, a type of rock consisting of consolidated volcanic ash ejected during a volcanic eruption. In such environments, radon concentration may be very high.

The occurrence of a wide spectrum of radon concentration in Italian caves, and the possibility to find some of these caves constantly inhabited by *Dolichopoda* cave crickets (Orthoptera, Rhaphidophoridae), led us to evaluate these insects as a suitable model to study the effects of radon on cave life.

*Dolichopoda* cave crickets are strictly dependent upon caves and several populations inhabit cave-like habitats, such as rock crevices and ravines, cellars, catacombs, aqueducts, Etruscan tombs and other similar man-made hypogean environments. They have long been studied in our laboratory from a wide array of genetic and ecological aspects addressed to understand their evolution and phylogeny [12, 13, 14, 15, 16, 17, 18, 19, 20, 21]. A preliminary study, carried out through the Comet assay, suggested a statistically significant dose-effect increase of DNA damage in specimens of *Dolichopoda* from radon-polluted caves, especially for the brain cells [22].

*Dolichopoda* populations and species have also been investigated for processes of molecular evolution of satellite DNA (satDNA), [23, 24, 25]. SatDNA is a class of non-coding DNA typically organized in large homogeneous arrays of tandemly arranged repetition units. These units are usually located in the heterochromatic parts of the chromosomes in the regions close to the centromeres and telomeres. Repeat size can vary largely within and between species from only a few base pairs up to several thousand base pairs [25 and references therein]. Three specific satDNA families have been characterized for *Dolichopoda* species, two of them being species-specific (*pDo102* and *pDsPv400*) and one (*pDo500*) occurring in all *Dolichopoda* species [23, 24]. A potential hammerhead (HH) ribozyme is embedded within the *pDo500* tandemly repeat satDNA [26, 27].

In the present study, we explored whether increasing level of radon-related ionizing radiation could induce an increased mutation pressure at the level of the *pDo500* tandemly repeat satDNA. We considered two species, *D*. *laetitiae* (Menozzi 1920) and *D*. *geniculata* (Costa, 1860) that, as demonstrated in previous studies [13, 14, 15, 18, 19], are genetically closely related. Population samples were collected in caves showing various amount of radon concentration, in order to investigate the possible association between radon-related ionizing radiation and the level of polymorphism in the *pDo500* tandemly repeat satDNA

## Materials and Methods

**Fig 1:**
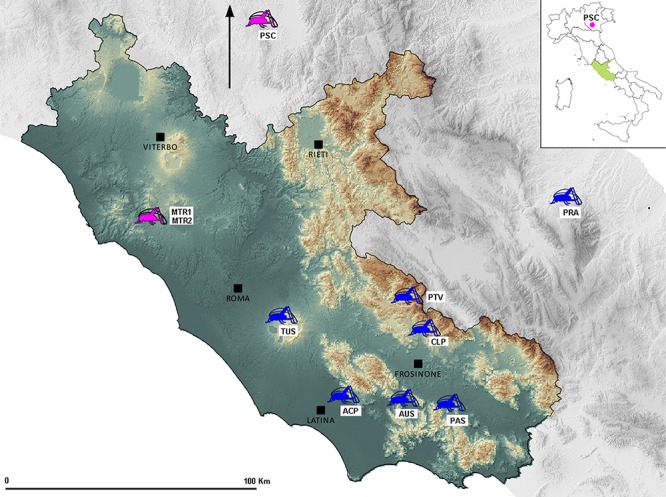
Sampling sites of *Dolichopoda* populations considered in this study (see also Table 1). Different colors refer to different species: pink to *D*. *laetitiae* and blue to *D*. *geniculata*.

This study was formerly approved by Regione Lazio in Italy (Dipartimento del Territorio), as a contribution to the knowledge of radon effects on insects constantly subjected to radioactivity. None of the field surveys in the present study involved endangered or protected species and no permission was necessary for the studied areas. Specimens for the DNA analysis were collected in seven different caves showing different radon concentration (Table 1, Fig 1). Sequences of *pDo500* tandemly repeats satDNA were derived from samples belonging to two populations of *D*. *laetitiae* and to five populations of *D*. *geniculata*. We also retrieved *pDo500* satDNA sequences from GenBank for population samples coming from other three caves whose radon’s concentration values were known: two population samples of *D*. *geniculata*, [25, 28] and one of *D*. *laetitiae* [25, 29]. See Table 1 for details.

**Table 1:** *Dolichopoda* population samples included in this study.

Species	Code	Locality	Average hourly concentration Bq/m^3^ ± SE	Sample size / Reference	GenBank Accession numbers
*D*. *laetitiae*					
	MTR1	Sulfur mine, Canale Monterano, Roma, Latium. Latitude: 42°6'47"; Longitude:12°27'47"	25997 ± 520	7 individuals, 17 clones / Present paper	KM598457- KM598475
	MTR2	Cellar, Canale Monterano, Roma, Latium. Latitude: 42°6'47"; Longitude:12°27'4"	2677 ± 134	12 individuals, 95 clones / Present paper	KM598476- KM598570
	PSC	Poscola cave, Priabona, Vicenza, Veneto. Latitude: 45°39'02"; Longitude:11°21'42"	200	4 individuals, 4 clones / (23, 29)	GU322289-GU322292
*D*. *geniculata*					
	PRA	Praie Cave, Lettomanoppello, Pescara, Abruzzo. Latitude: 42°14'22"; Longitude: 14°3'18"	982	5 individuals, 5 clones / (23, 28)	GU322284-GU322286
	PTV	Pertuso di Trevi Cave, Filettino, Frosinone, Latium. Latitude:41°87.11'81.03"; Longitude: 13°28.22'84.43"	13200	10 individuals, 32 clones / (28)	KP399737-KP399768
	TUS	Tuscolo Cave, Roman Aqueduct, Frascati, Latium. Latitude: 41°47'57.37"; Longitude: 12°41'47.25"	1906 ± 76	7 individuals, 11 clones / (23 and Present paper)	GU322316-GU322319KM598585- KM598591
	ACP	Fiume Coperto Cave, Sermoneta, Latina, Latium. Latitude: 41°51.74'49.56"; Longitude: 12°99.38'41.14"	1305 ± 91	3 individuals, 7 clones / Present paper	KM598447- KM598453
	AUS	Ausi Cave, Prossedi, Latina, Latium. Latitude: 41°50.99'20.72"; Longitude:13°27.41'24.99"	1047±379	5 individuals, 5 clones / (23, 28)	GU322149- GU322153
	CLP	Regina Margherita Cave, Collepardo, Frosinone, Latium. Latitude: 41°75.91'53.45", Longitude:13°36.71'19.97"	221 ± 35	6 individuals, 8 clones / (23 and Present paper)	GU322194-GU322199KM598454- KM598456
	PAS	Pastena Cave, Pastena, Frosinone, Latium. Latitude: 41°49.76'23.9"; Longitude: 13°49.10'18.89"13,4910188941,4976239	2385 ± 119	10 individuals, 19 clones / (23 and Present paper)	GU322261-GU322265KM598571- KM598584

Radon radioactivity measurements (Bq/m^3^) and GenBank Accession numbers are also reported.

### Radon measurements

The presence of radon in caves was detected by the Alfa track detector LR115. This detector has a particular film capable of measuring ^222^Rn concentration. Its working is based on the principle that the radon's alpha particles leave traces on a film coated with a thin layer of gelatin. It has to be placed in a stable and dry location for an adequate time. In this study, the Alfa track detector LR115 was located at the center of the cave in all considered sites except for the Pastena cave (PAS). The latter is a large cave subdivided in two distinct rooms and the specialized equipment was located at the center of each room. Following the manufacturer instructions, the Alfa track detector was left in the caves for a month, to obtain the measure of the average concentration of radon based on traces left by alpha particles (certificate numbers from 13843 to 13849, in accordance with U.S EPA National Radon Proficiency Program EPA—CFA Recommended Test Report Format).

### Laboratory procedures

Genomic DNA was extracted from leg muscles using the Sigma-Aldrich GenElute Mammalian genomic DNA Miniprep Kit, following the instructions.

*PDo500* satDNA sequences were amplified with the following primers, 5'-GTTTTACACGTTCACTGCAG-3' and 5' GACACATTGATGAGACTGCAG-3' [24]. The PCR conditions were as follow: 95°C for 3 minutes, followed by 30 cycles of denaturation at 95°C for 30 seconds, annealing at 50°C for 30 seconds, elongation at 72°C for 30 seconds and one final elongation step at 72°C for 2 minutes. The obtained PCR products were cloned using the pGEM-T Easy Vector kit (Promega). Positive clones were selected through PCR amplification using the reverse and forward M13 primers. The obtained PCR products were purified using the enzymatic digestion (ExoSAP-IT, Affymetrix, U.K.) and sequenced using the ABI-3730 Genetic Analyzer. Alignment was carried out using Clustal X 1.81 [30].

### Data analyses

SatDNA repeat polymorphism, considering the estimates of nucleotide diversity (π) and the average number of nucleotide differences (K), was investigated using DNAsp software [31] for each sampled population. DNAsp was also used to perform sliding window analysis in order to detect regions of high sequence conservation. We carried out this analysis by considering sites with alignment gap in the window length. The window size was set to 30 with step size of 5. The analysis was performed on both the complete alignment for each population and on consensus sequences for each species.

Insertion-deletion polymorphism was also analyzed, using the multiallelic option in DNAsp. The total number of indel events, the average indel length per event, the number of indel haplotypes and the indel haplotype diversity were calculated for each population.

To investigate possible relations among the radon concentration in caves, the amount of satDNA polymorphism, and possibly the taxonomic status of each population, two type of multivariate analyses were carried out. In particular, multivariate ordination of *Dolichopoda* population samples based on polymorphism’s measures was studied by Factorial Correspondence Analysis (FCA), [32], using xlstat 2014. The radon concentration in caves and the taxonomic status of each population were considered as supplementary variables. In this way, these two variables were not taken into account for the computation of the representation space and their coordinates were computed a posteriori.

A multivariate multiple regression analysis (manova) was carried out using past software [33] to compare radon concentration in caves and the taxonomic status of each population with the measures of satDNA polymorphism. In particular, the frequency of polymorphic sites (PSF) and indel sites, the average length of indel and the haplotype diversity per population were log transformed and considered in this analysis. In order to exclude the possibility that our results were influenced by the unbalanced sample size, we carried out a Linear Mixed Model, using xlstat 2014, considering the environmental radioactivity as explanatory variable and the length of indel, calculated for each individual, as the response variable. Radioactivity measures (Bq/m^3^) were log transformed and considered as fixed effect. To test the non-independency of data from the same cave, each cave population was considered as a random effect. Likelihood ratio tests (LRT) were computed using the restricted maximum likelihood (REML) method, as implemented in xlstat 2014. The significance of the fixed effect was tested by a LRT between the full model and a null model comprising only the intercept and the random effects.

## Results

### Radon measurements

Table 1 shows the mean values of natural radioactivity for each of ten measured caves. The highest levels of radioactivity were reported in MTR1 and PTV caves, showing values of 25,997 and 13,200 Bq/m^3^, respectively. All other caves, except for CLP and PSC, showed ^222^Rn concentration measures ranging between 982 and 2,700 Bq/m^3^, which are much higher than the lowest threshold value established by European Union for the radon concentration in dwellings (400 Bq/m^3^). Only CLP and PSC caves exhibited radon concentration levels close to the threshold established for human housing.

### Satellite DNA

We analyzed 163 satDNA repeats of the *pDo500* family from seven sampled populations, each represented with 4–95 sequences. The length of the *pDo500* sequences ranged between 458 and 481 bp. The total alignment consisted of 500 positions. The average nucleotide composition was T = 35.2%, C = 23.9%, A = 21.6% and G = 19.3%. The estimated transition/transversion bias (R) was 0.79.

**Fig 2:**
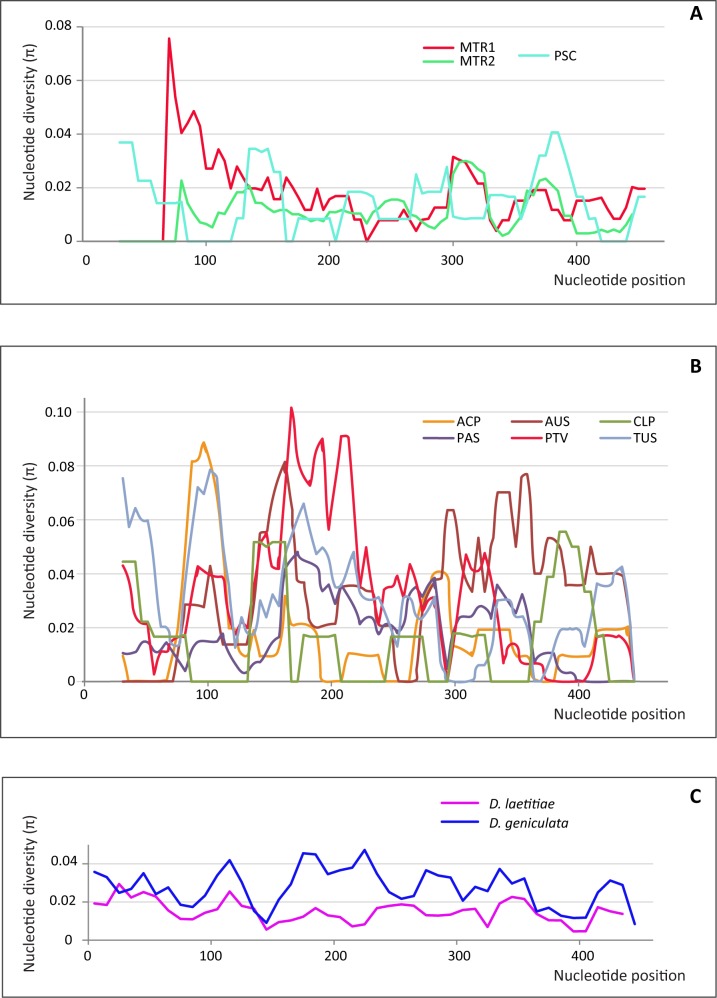
Sliding window analyses of the *pDo500* satellite DNA in populations of *D*. *laetitiae* and *D*. *geniculata* included in this study. The analysis was performed for each population of *D*. *laetitiae* (A), *D*. *geniculata* (B) and on consensus sequences for each species (C). The value of nucleotide diversity (π) was obtained by a sliding window size of 30 with step size 5.

Results from sliding window analyses, carried out separately for each population and each species, indicated that population samples coming from caves with the highest radioactivity showed also the highest nucleotide diversity in the peaks of local maxima, regardless of the species (MTR1 in *D*.*laetitiae* and PTV in *D*.*geniculata*, Fig 2A and 2B). Results from sliding window analyses carried out on consensus sequences for each species (Fig 2C) indicated higher nucleotide diversity in *D*. *geniculata* (π ranged from 0 to 0.1) than in *D*. *laetitiae* (π ranged from 0 to 0.07).

Table 2 shows the estimates of *pDo500* satDNA polymorphism within population samples. Frequency of polymorphic sites (PSF), frequency of indel sites (SIF), and average length of indel (ALI), are remarkably higher in population samples coming from the three caves, with the highest radioactivity measures: MTR1 and MTR2 housing *D*. *laetitiae* and PTV housing *D*. *geniculata*. One specimen from MTR1, five from MTR2 and five from PTV showed also clones with very short sequences ranging from 102 to 378 bp. These short sequences were characterized by a large gap in the middle of the *pDo500* satDNA sequence. In particular, deletion covered the region between the first 10 bases of *pDo500* and the position 296 in the alignment and did not overlap the region of the potential hammerhead (HH) ribozyme embedded within the *pDo500* satDNA [25; 26]. These short sequences were taken into account only when indel polymorphism analysis was carried out.

**Table 2:** Polymorphism estimates in sampled populations of *Dolichopoda* cave crickets.

Population	PSF	SIF	ALI	HIF	HTF	HID	HTD ± Stand. Dev.	K± Stand. Dev.	π ± Stand. Dev.
MTR1	0.125	0.635	44.000	0.263	0.944	0.526	0.993 ± 0.021	8.719 ± 0.585	0.020 ± 0.003
MTR2	0.283	0.760	57.625	0.179	0.648	0.344	0.962 ± 0.013	4.838 ± 0.190	0.012 ± 0.001
PSC	0.033	0.009	2.000	0.750	1.000	0.833	1.000 ± 0.177	8.000 ± 1.333	0.018 ± 0.003
PRA	0.073	0.045	2.333	0.800	1.000	0.900	1.000 ± 0.126	18.200 ± 1.742	0.041 ± 0.009
PTV	0.122	0.645	18.765	0.656	0.815	0.972	0.988 ± 0.016	13.160 ± 0.592	0.030 ± 0.003
TUS	0.134	0.028	1.300	0.727	1.000	0.891	1.000 ± 0.039	14.782 ± 0.992	0.033 ± 0.005
ACP	0.055	0.002	1.000	0.286	0.857	0.286	0.952 ± 0.096	8.000 ± 0.943	0.017 ± 0.004
AUS	0.068	0.013	1.200	0.800	0.800	0.900	0.900 ± 0.161	14.100 ± 1.533	0.031 ± 0.006
CLP	0.068	0.007	1.000	0.375	0.875	0.607	0.964 ± 0.077	9.321 ± 0.942	0.020 ± 0.003
PAS	0.139	0.065	3.333	0.474	0.895	0.778	0.988 ± 0.021	9.462 ± 0.592	0.022 ± 0.003

PSF: Frequency of polymorphic sites; SIF: Frequency of indel sites; ALI: Average length of indel; HIF: Frequency of indel haplotypes; HTF: Haplotype frequency; HID: Indel haplotype diversity; HTD: Total haplotype diversity; K: Average number of nucleotide differences; π: Nucleotide diversity. Standard Deviations are also reported for HTD, K and π.

Multiple regression analysis (manova) revealed a significant correlation between the polymorphism estimates and the radon concentration in cave (F = 4.02, Wilk’s lambda = 0.040; P = 0.033). In particular, the frequency of indel sites (SIF, p = 0.008) and the average length of indel (ALI, p = 0.004) were statistically significant correlated with the levels of radioactivity in cave. On the other hand, the nucleotide diversity (K, p = 0.057) and the average number of nucleotide differences (π, p = 0.078) showed a high tendency to be dependent from the taxonomic status of each population.

Results obtained from LMM analysis, carried out for each individual, showed a significant regression line (P = 0.05; Table 3), indicating an increase of the length of indel related to the radioactivity levels measured in the caves.

**Fig 3:**
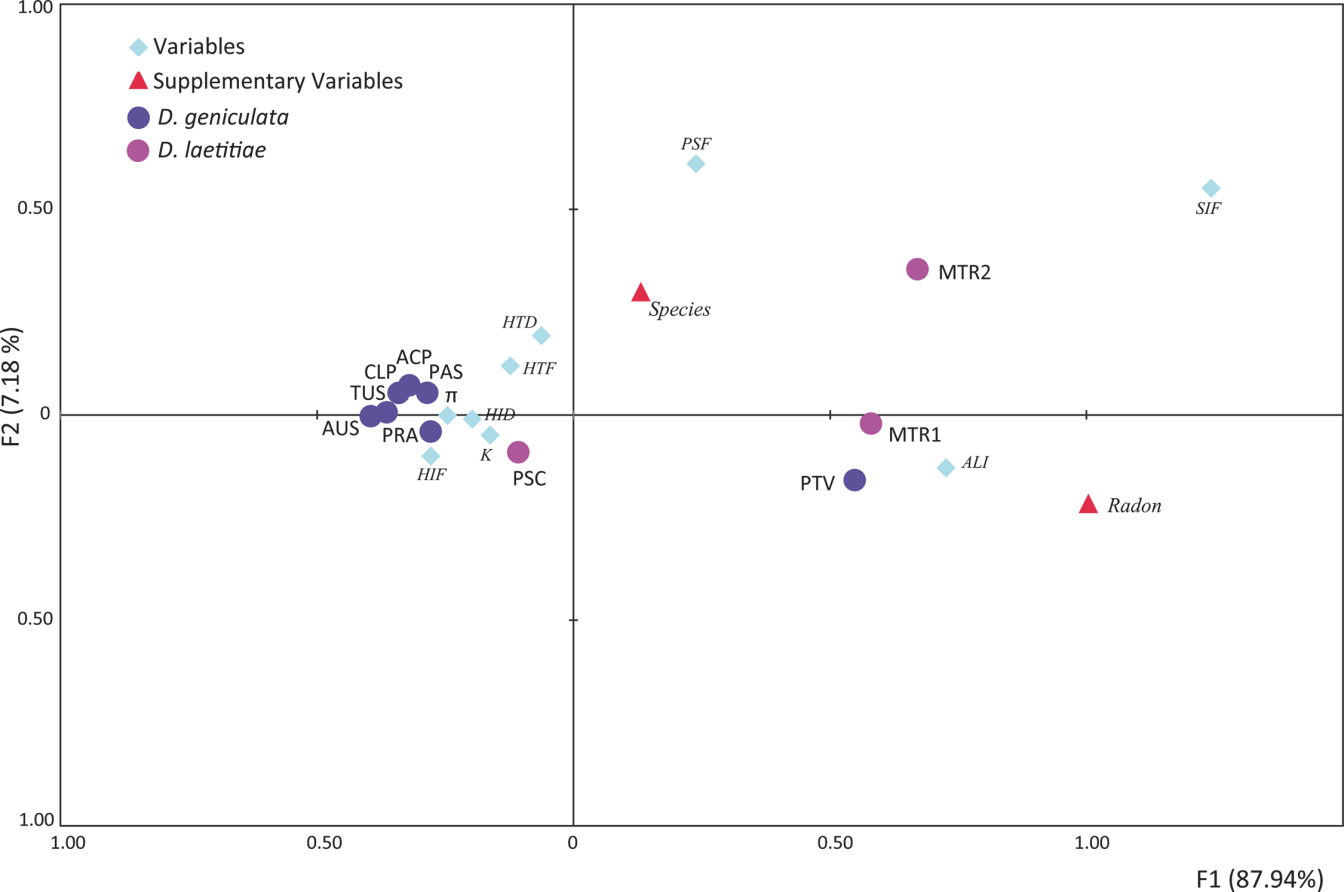
Factorial Correspondence Analysis on polymorphism measures of *Dolichopoda* populations analyzed in this study. Ordination of populations on the plane is described by the first two axes. Population samples coming from caves with high radon concentration are displaced into different portions of the ordination plane.

Fig 3 reports results from FCA with the first two axes explaining together 95.12% of the total variance. The first axis clearly separates population samples subjected to high radioactivity from all the others.

**Table 3:** Estimated values, standard errors (*SE*), *t*-values and significance (*P*-values) obtained by Likelihood Ratio Test (LRT) of full vs. reduced models, for the coefficients of fixed effects in Linear Mixed Models (LMMs) for the average length of indel (ALI, see text for details).

Fixed effect	Estimate	*SE*	*t*-value	LRT *df*	LRT *χ2*	*P*-value
Intercept	-50.571	36.769	-1.375			
*log* radioactivity	20.168	10.377	1.944	1	13.453	0.053

## Discussion

In recent years, the impact of chemicals and physical pollutants on the functionality of DNA has been investigated in many animal species [34]. Most of the studies evaluated the biological response to the agents considering gene mutation, chromosome aberration, sister chromatid exchanges, DNA damage by Comet assay, micronuclei [22, 35, 36, 37]. In this study, the possible biological response to the environmental radioactivity was investigated by considering a specific category of nuclear DNA, the satDNA, and two genetically closely related species. We used two species, in order to verify if, regardless of the specific genetic variability, they could have the same biological response to the environmental contaminant.

The analysis of variability showed that *D*. *geniculata* is more polymorphic than *D*. *laetitiae*, as expected [25]; (Table 2, Fig 2). However, we found significant correlations between some polymorphism estimates and radon concentration in caves in both species, regardless of the degree of variability expressed by each one. In particular, both manova and FCA analyses (Fig 3) revealed that the indel polymorphism (SIF, ALI; Table 2) is significantly correlated with radon concentration (Bq/m^3^; Table 1); while the nucleotide diversity and the average number of nucleotide differences appear to be species dependent. In particular, the two localities MTR1, hosting *D*. *laetitiae*, and PTV, hosting *D*. *geniculata*, showed ^222^Rn levels higher of one to two orders of magnitude than the other caves (Table 1). This very high radioactivity can be explained by the presence of sulfur springs in MTR1 and by both the very low circulation of air and the presence of numerous faults and fractures in PTV. Samples coming from these two sites showed an average length of indels (ALI; Table 2) greater than that observed in other caves. High levels of *pDo500* polymorphism (SIF, ALI, PSF; Table 2) were detected also in *Dolichopoda* samples from MTR2 site, although the latter showed a radioactivity level of one order of magnitude lower than in MTR1 and PTV caves (Table 1). This result might be explained by assuming that samples from MTR1 and MTR2 belong to the same population. Indeed, these localities are very close and are located in the Natural Reserve of Monterano, MTR1 is an old sulfur mine, MTR2 is a cellar in an old ruined house built with tuff and is located at 100 meters away in front of MTR1, being separated by the Mignone river. The two caves are surrounded by woods, an optimal environment for *Dolichopoda* that, at night, exit the cave to forage and move around. Results from mtDNA Cytochrome Oxidase I sequences showed that individuals coming from the one or the other site are very similar, showing at most a singleton difference (not published data) and *Dolichopoda* cave crickets show generally a certain degree of mtDNA variability both between and within populations [19, 20, 21]. Therefore, it is reasonable to consider these two sites as hosting a single population and to expect that individuals of *Dolichopoda* transfer from one cave to another, being definitely subjected to the same radon dose.

The LMM analysis, carried out for each individual to attempt to correct for our unequal sample size, confirmed these results (Table 3), suggesting, again, that the radioactivity levels measured in the caves appear to be responsible for the gaps’ length observed in our samples.

The biological significance of satDNA has been the object of several discussion and generally, based on the diversity of satDNA in nucleotide sequences, length of repeats, genomic abundance, a specific function has not been yet assigned to this genomic region. However, a number of possible functions have been hypothesized [38] and most of them are related to heterochromatin and/or centromere formation and function.

Samples from populations in hypogean environments with the highest radioactivity showed also the highest frequency of indel sites and clones with *pDo500* repeat sequences shorter than the standard, but that are integer at level of HH ribozyme region. Previous studies suggested that the HH region of the *pDo500* sequence family has a functional role in *Dolichopoda* cave crickets although its function is unclear and remains to be investigated [27]. Our data seem to support the hypothesis that the HH ribozyme region could have an important role, since this region is never affected by the events of insertion / deletion.

Finally, our results are consistent with those from [22] where six out of the ten populations here studied were analyzed for DNA primary damage through Comet assay. Both haemocytes and brain cells taken from individuals from radon-polluted caves were tested and compared to a control group of cave crickets reared in absence of radon. Results indicated a statistically significant dose-effect increase of DNA damage in all caves, especially for the brain cells. In conclusion, we can infer from present data that the increased sequence heterogeneity at *pDo500* satDNA level can be explained as an effect of the mutation pressure induced by radon in cave. Furthermore, we discovered a specific category of nuclear DNA, the highly repetitive satDNA sequences, where the effects of the exposure at high levels of radon-related ionizing radiation are detectable. Future researches could be addressed to evaluate and investigate if satDNA might be a valuable tool to reveal the effects of radon in other organisms.
